# Supplementary material for: Genomic analyses of Mycobacterium tuberculosis from human lung resections reveal a high frequency of polyclonal infections
Source: Nat Commun. 2021 May 11;12:2716. doi: 10.1038/s41467-021-22705-z (PMC8113332; doi:10.1038/s41467-021-22705-z)
Supplement: Supplementary file 1 — Supplementary Information [file 41467_2021_22705_MOESM1_ESM.pdf]

SUPPLEMENTARY INFORMATION

Supplementary Note 1: Additional details on surgical patients

When analyzing the surgical patient dataset, we first hypothesized that if there are gradients of drug penetration across the lesion as reported in the literature, this may translate into differences of MTB genomic diversity across these positions in and around TB lesion, and that these differences will be observed both in drug resistance associated genes as well as across the whole MTB genome. As shown in Supplementary Figures 2 and 3, there are important differences in terms of diversity among patient samples. In patient G018, most of the MTB diversity accumulated in the granuloma center. When focusing on exclusive SNPs, most of these were located in the center of the lesion (127/215 SNPs or 59%). The caseum was mainly dominated by variants at 3-5% frequency, suggesting that these were relatively recent. By contrast, the rest of the samples, including the sputum, showed a lower number of low frequency variants, probably reflecting population bottlenecks. In fact, the principal component analysis for this patient (Supplementary Figure 2C) shows that the diagnostic sputum sample was closer to the MTB diversity seen in the sample from the nearby healthy tissue. Additionally, the remote nodule from this patient did not share many of the mutations found in the other samples, which however, may be partially explained by a lower sequencing depth. For this patient, we were also interested in knowing how well the MTB diversity in the sputum sample was represented by the variants in the surgical samples. For this analysis, we pooled all low frequency variants identified in the surgical samples and compared them to the sputum sample. Only 13 out of the 461 (2.8%) low-frequency variants seen in the sputum were not present in any of the surgical samples. Twelve of these thirteen variants were at 3-5% frequency, aside from one at 74%. In addition, we found some mutations arising in the center of the lesion in resistance-associated genes and not described before in drug resistance catalogs. These cavity center mutations were at very low frequencies in the sputum culture sample of the patient, well below common detection thresholds (Supplementary Figure 2B).

Some other interesting cases are patients G335, G324 or G033. In patient G335, we detected just one genotype and we could not find any drug resistance associated mutation. This is consistent with the phenotypic DST results of this patient. The PCA showed that the sputum sample was closer to the one from the cavity center, but the differences across sites were minimal and most of the variants were shared between samples (Supplementary Figure 2C). Patient G324 was another case of polyclonal infection, with a sputum sample harbouring a different strain from the one present in the other surgical samples. The PCA reflected this, showing surgery samples separating along PC2 and only the sputum isolate far away from them in PC1. We also did not find any trace of the sputum strain in the caseum when looking at low frequencies. Patient G033 was infected with two different L4 sublineages (G1=L4.3.3, G2=L4.2.1) in the nodule (90% G1, 10% G2) and healthy tissue (10% G1, 90% G2). The whole granuloma (C, I, E) was 100% G1 (Supplementary Figure 2A). This was also reflected in the PCA , where both of those samples did not cluster with the other samples, nor with each other. Remarkably, G1 was XDR while G2 was fully drug susceptible.

Supplementary Note 2: Genomic predictions vs DST accuracy evaluation

Predictions of the phenotype for first line drugs using sequencing data and available catalogs (see Methods) was overall accurate in this dataset (sensitivity 0.938, specificity 0.994, see Supplementary Figure 7). Most drug resistance-associated mutations for first line drugs were already fixed in the population (frequency > 90%) as expected for patients that have undergone surgery after failing their first line treatment. In addition, eight variants present in catalogs were below standard levels of detection (frequency < 10%), however they were always concomitant to other known variants for the same drug so they do not explain potential disagreements between genotype and phenotype. Genotype failed to predict some of the phenotypic DST results. These include one kanamycin, 5 ethambutol, 3 para-aminosalicylic acid, 2 streptomycin and 1 capreomycin discrepancies. We suggest that two new mutations (2747471AG, *folC* I43T; 3073679CA, intergenic *dfrA-thyA*) might explain PAS resistance not detected by available catalogues, as both of this genomic features have been described to be involved in the drug metabolism. Further studies would be needed to assess the role of these mutations. We were not able to pinpoint any mutations to explain the rest of discrepancies.

SUPPLEMENTARY TABLES

Supplementary Table 1. Details of serial sputa pairs dataset.

Patient	G1	G2	G1 Profile	G2 Profile	Distance	Days Diff.	Infection
**G-065**	L2.2.9	-	Pre-XDR	-	0	267	Clonal
**G-069**	L2.2.9	-	MDR	-	0	108	Clonal
**G-078**	L2.2.10	-	MDR	-	0	294	Clonal
**G-128**	L2.2.9	L1.1.1.1	MDR	MDR	1740	318	Polyclonal
**G-169**	L4.3.3	L2.2.9	Susceptible	XDR	1185	352	Polyclonal
**G-205**	L2.2.9	-	MDR	-	0	2	Clonal
**G-208**	L2.2.10	-	Pre-XDR	-	1	244	Clonal
**G-209**	L4.8	L2.2.10	Susceptible	Pre-XDR	1168	101	Polyclonal
**G-213**	L2.2.10	-	Pre-XDR	-	0	595	Clonal
**G-214**	L2.2.10	-	Pre-XDR	-	0	57	Clonal
**G-215**	L2.2.9	-	Pre-XDR	-	0	190	Clonal
**G-216**	L2.2.10	-	Poly-res	-	2	91	Clonal
**G-217**	L2.2.10	-	Pre-XDR	-	0	247	Clonal
**G-219**	L2.2.9	-	XDR	-	1	28	Clonal
**G-220**	L2.2.9	L2.2.9	Pre-XDR	-	10	431	Clonal
**G-221**	L4.3.3	-	MDR	-	0	62	Clonal
**G-223**	L4	L2.2.9	MDR	XDR	1128	333	Polyclonal
**G-224**	L2.2.10	L2.2.10	Pre-XDR	Pre-XDR	1105	38	Polyclonal
**G-225**	L2.2.9	-	MDR	-	0	41	Clonal
**G-227**	L2.2.10	-	Mono-INH	-	1	92	Clonal
**G-229**	L4.6.2	-	Susceptible	-	0	31	Clonal
**G-230**	L4.8	-	Susceptible	-	0	13	Clonal
**G-246**	L4.2.1	L2.2.10	Pre-XDR	XDR	811	24	Polyclonal
**G-250**	L2.2.10	-	MDR	-	0	41	Clonal
**G-255**	L2.2.9	-	MDR	-	0	75	Clonal
**G-258**	L4.8	-	Susceptible	-	2	125	Clonal
**G-262**	L2.2.10	-	Pre-XDR	-	0	95	Clonal
**G-270**	L2.2.10	-	Pre-XDR	-	0	97	Clonal
**G-275**	L2.2.9	-	MDR	-	0	45	Clonal
**G-276**	L2.2.10	-	MDR	-	0	39	Clonal
**G-278**	L2.2.10	-	XDR	-	3	122	Clonal
**G-280**	L2.2.10	-	Pre-XDR	-	0	3	Clonal
**G-281**	L2.2.9	-	MDR	-	1	123	Clonal
**G-284**	L2.2.9	-	Pre-XDR	-	0	70	Clonal
**G-285**	L4.2.1	-	MDR	-	0	266	Clonal
**G-287**	L2.2.10	-	MDR	-	0	68	Clonal
**G-297**	*	*	*	*	*	117	Polyclonal
**G-315**	L2.2.10	-	Susceptible	-	0	1	Clonal

* G297 was a special case in which both sputum samples had three different genotypes at once (L4.3.3, L4.8 and L2 for S1; L4.3.3, L2.2.9 and L2.2.10), but only one of them (L4.3.3) was a match, making it complex to establish the distance between each one of them.

SUPPLEMENTARY FIGURES

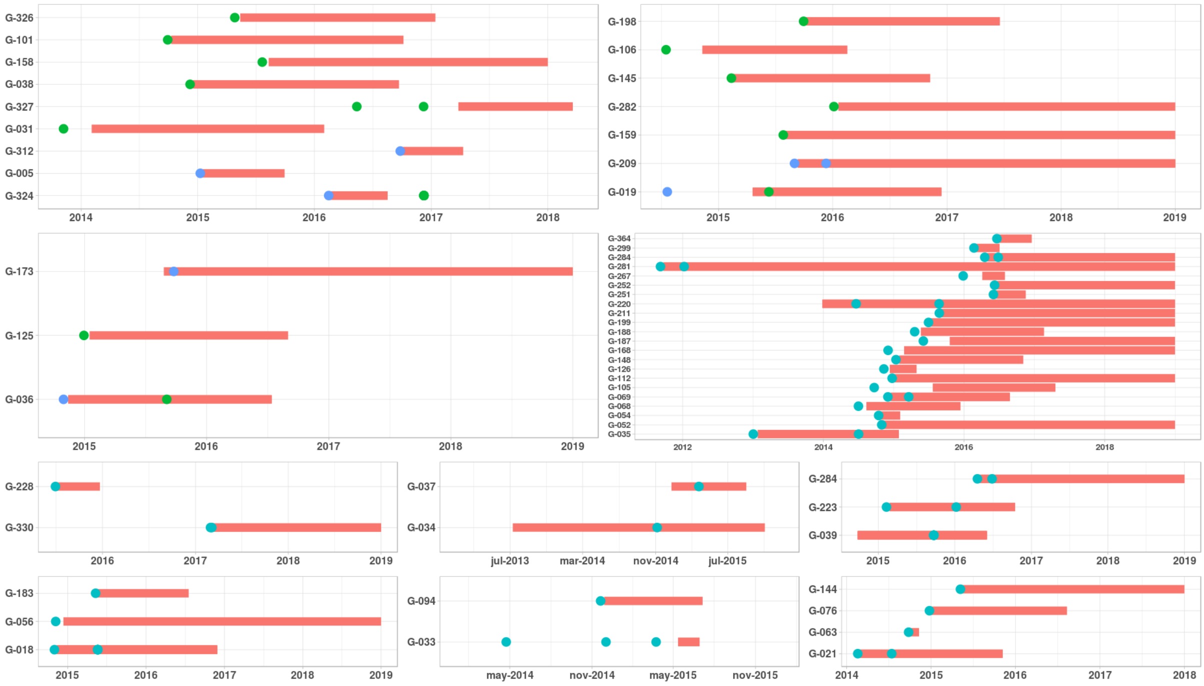

Supplementary Figure 1. Timeline of surgical patients involved in transmission clusters. The surgical patient is always depicted at the bottom. If the paired samples belong to different clusters, they are represented in two colors and patients of those two clusters are in the same graph identified with colors (blue = sputum cluster, green = surgical cluster).

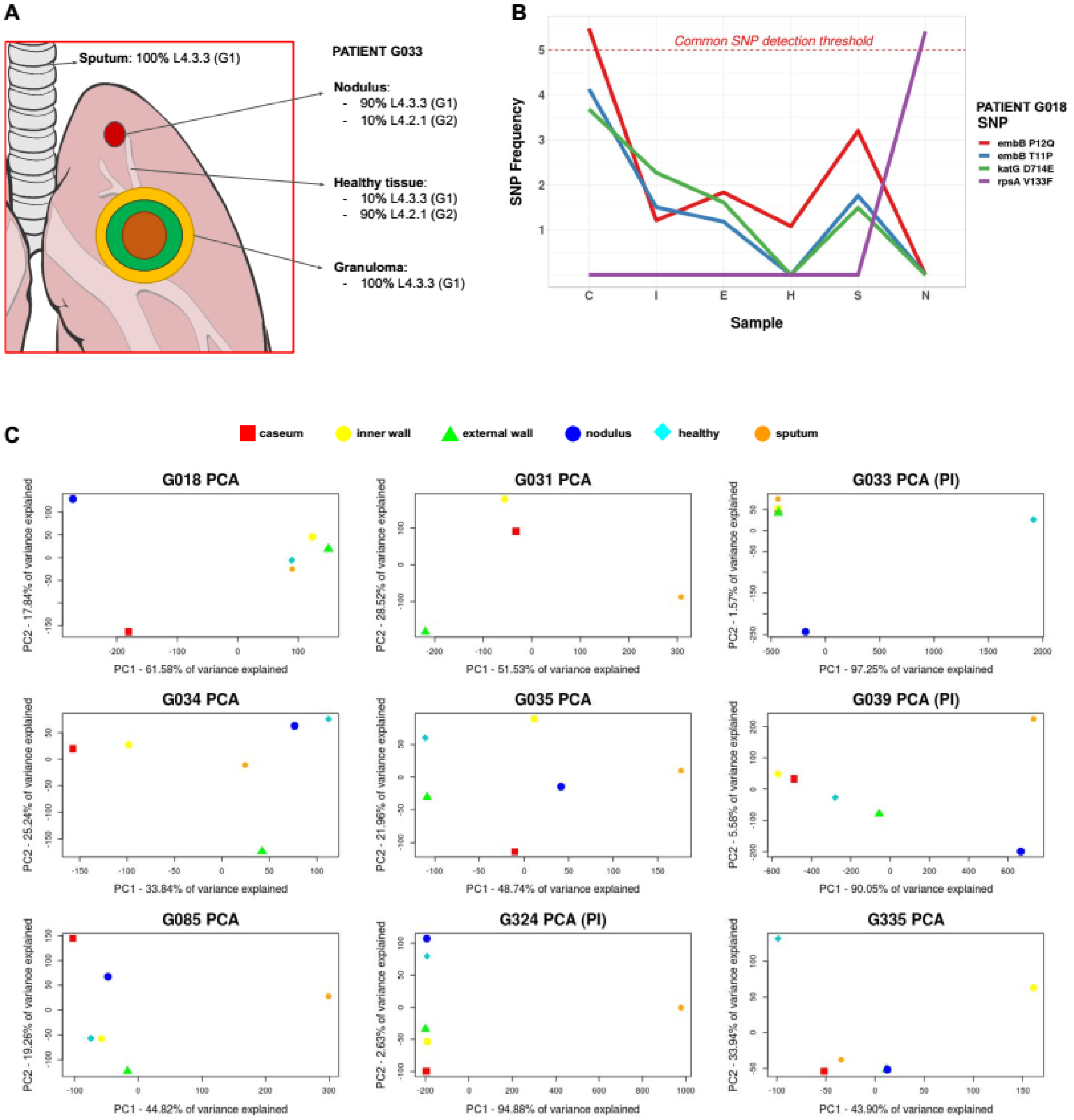

Supplementary Figure 2. Additional details on the surgical cohort. A) Details on G033 genotype distribution. B) Example of a set of SNPs in resistance-related genes that are originally above 3% frequency in the caseum sample and how we can trace their frequencies across the rest of the lesion. As we validate those variants as not spurious, it is highly likely that their detection in samples below 3% is accurate. C) PCA analysis for all nine multi-sample surgical patients. Abbreviations: G1, genotype 1; G2, genotype 2; PI, polyclonal infection.

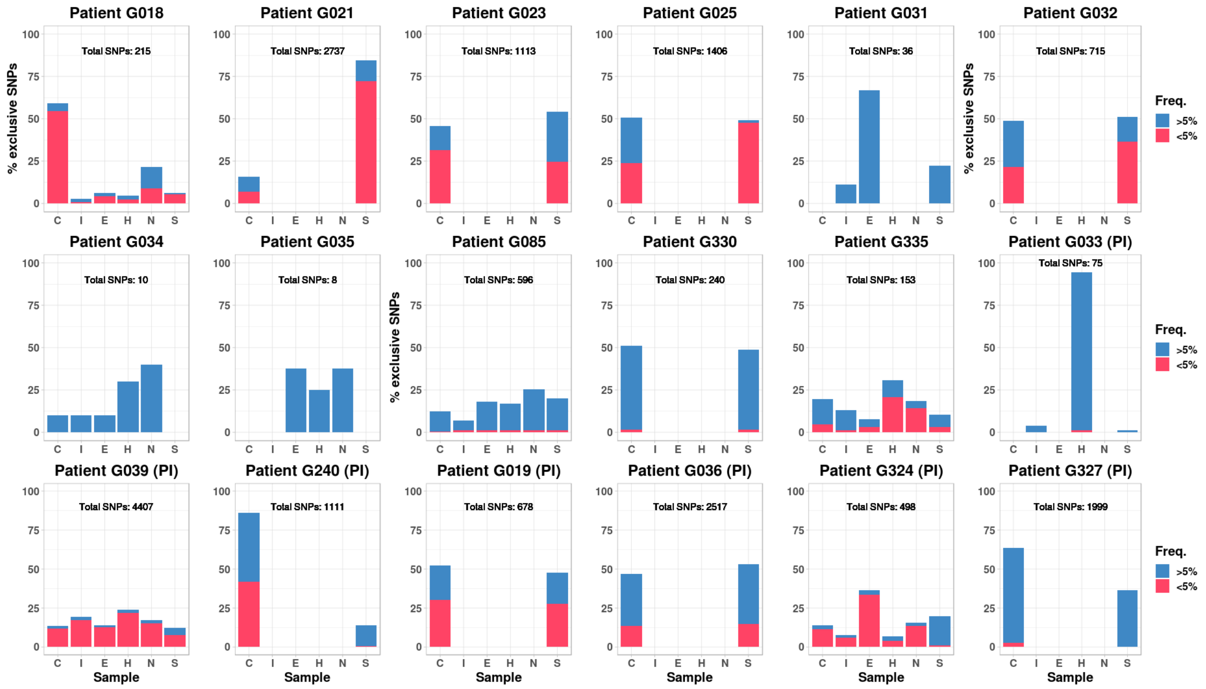

Supplementary Figure 3. Exclusive SNPs among isolates of surgery patients. Bars represent the percentage of the total private SNPs, also noted in each individual graph. Each sample type is divided into blue (SNP freq. >5%) and red bars (SNP freq. 3-5%). Abbreviations: PI, polyclonal infection.

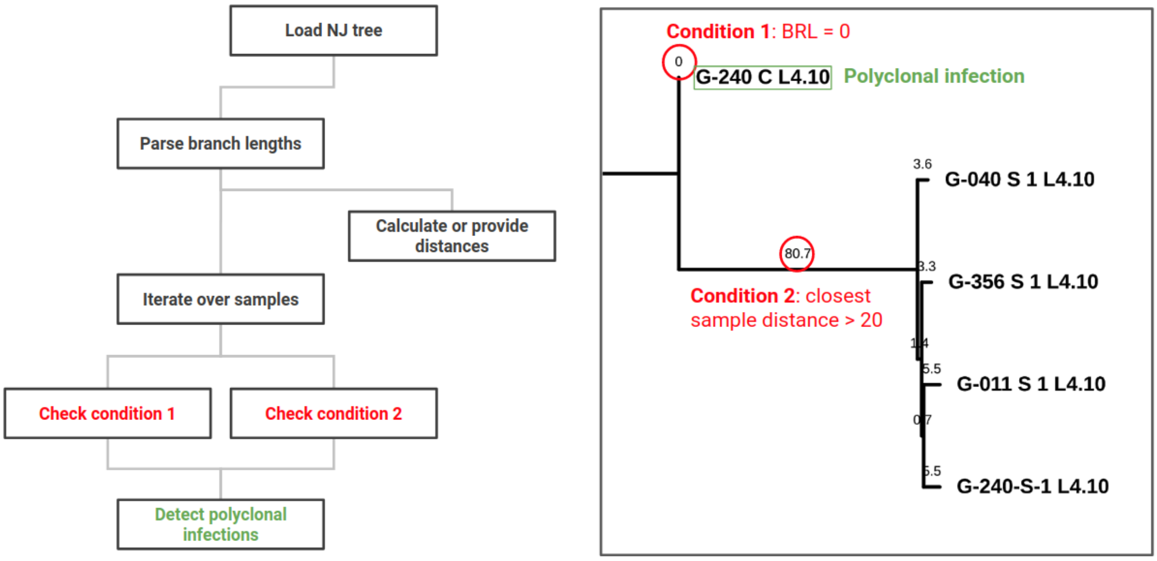

Supplementary Figure 4. Flowchart of phylogenetic identification of polyclonal infections. A Python script loads a NJ tree and scans for isolates that show terminal branch length equal to zero and distance to other isolates higher than 20 fixed SNPs. An example for one of the surgical patients is provided: the caseum sample from patient G240 is a mix of their sputum genotype and G040.

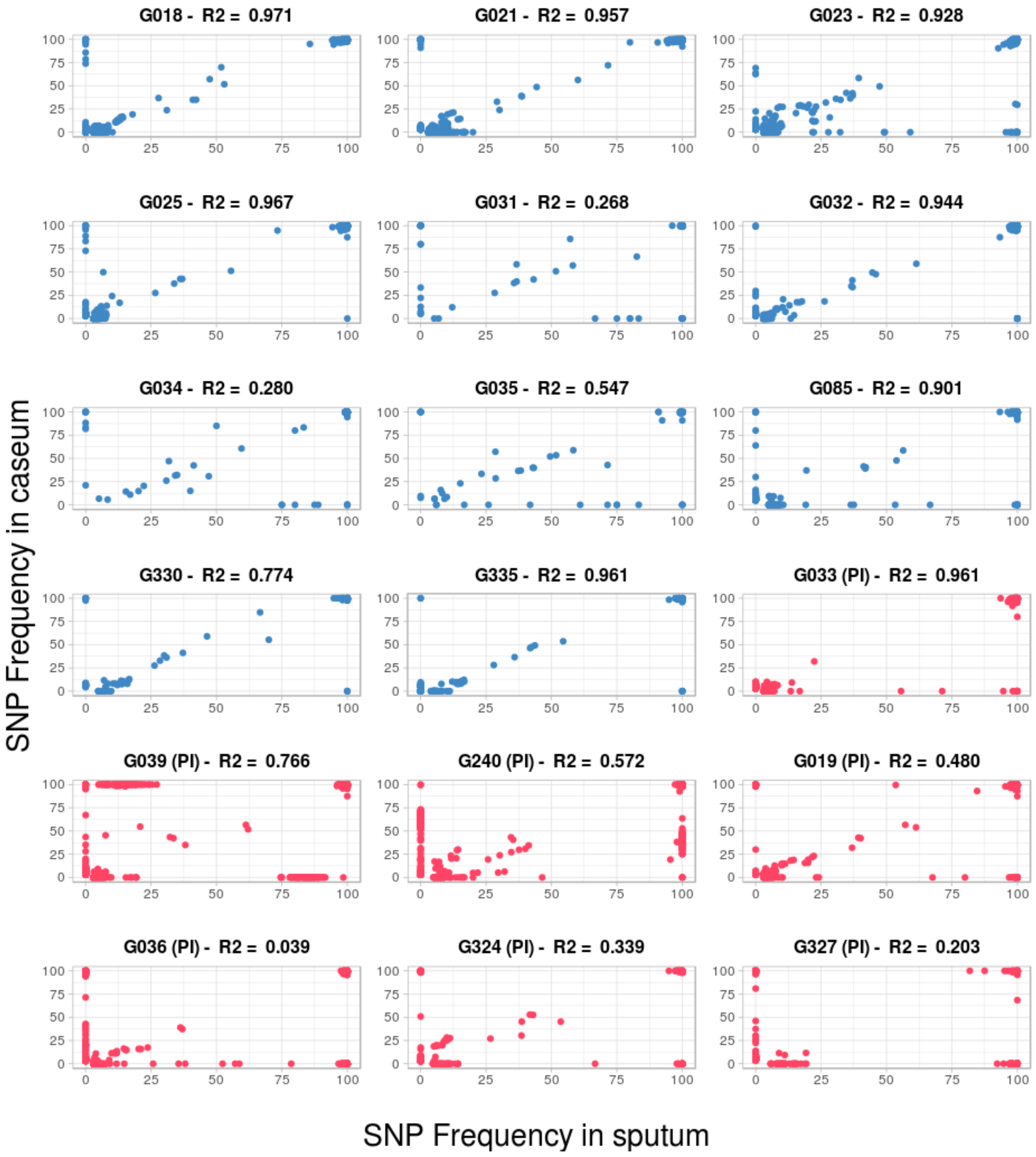

Supplementary Figure 5. Correlation between sputum and caseum SNP frequencies for all surgery patients. Blue graphs correspond to clonal infections and red graphs to polyclonal infections. R^2 usually decreases when multiple genotypes are involved in an infection. Abbreviations: PI, polyclonal infection.

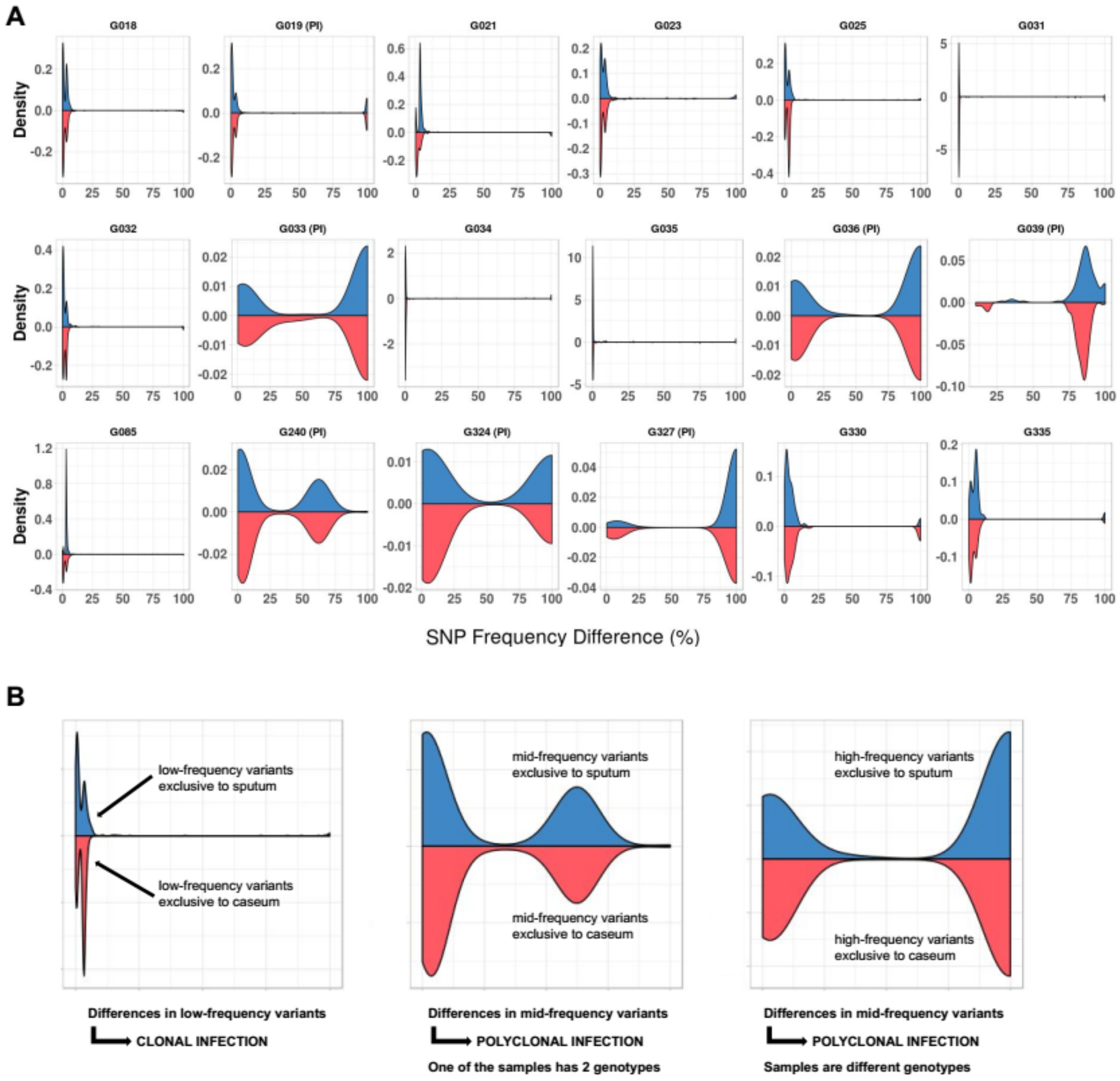

Supplementary Figure 6. Density profiles of SNP frequency differences between sample pairs (sputum in blue, caseum in red) in the surgery cohort. A) Different patterns reflect clonal infections (all differences in very low frequency range) or polyclonal infections (differences found in middle and/or high frequencies). B) Details on how to interpret the density graphs. Abbreviations: PI, polyclonal infection.

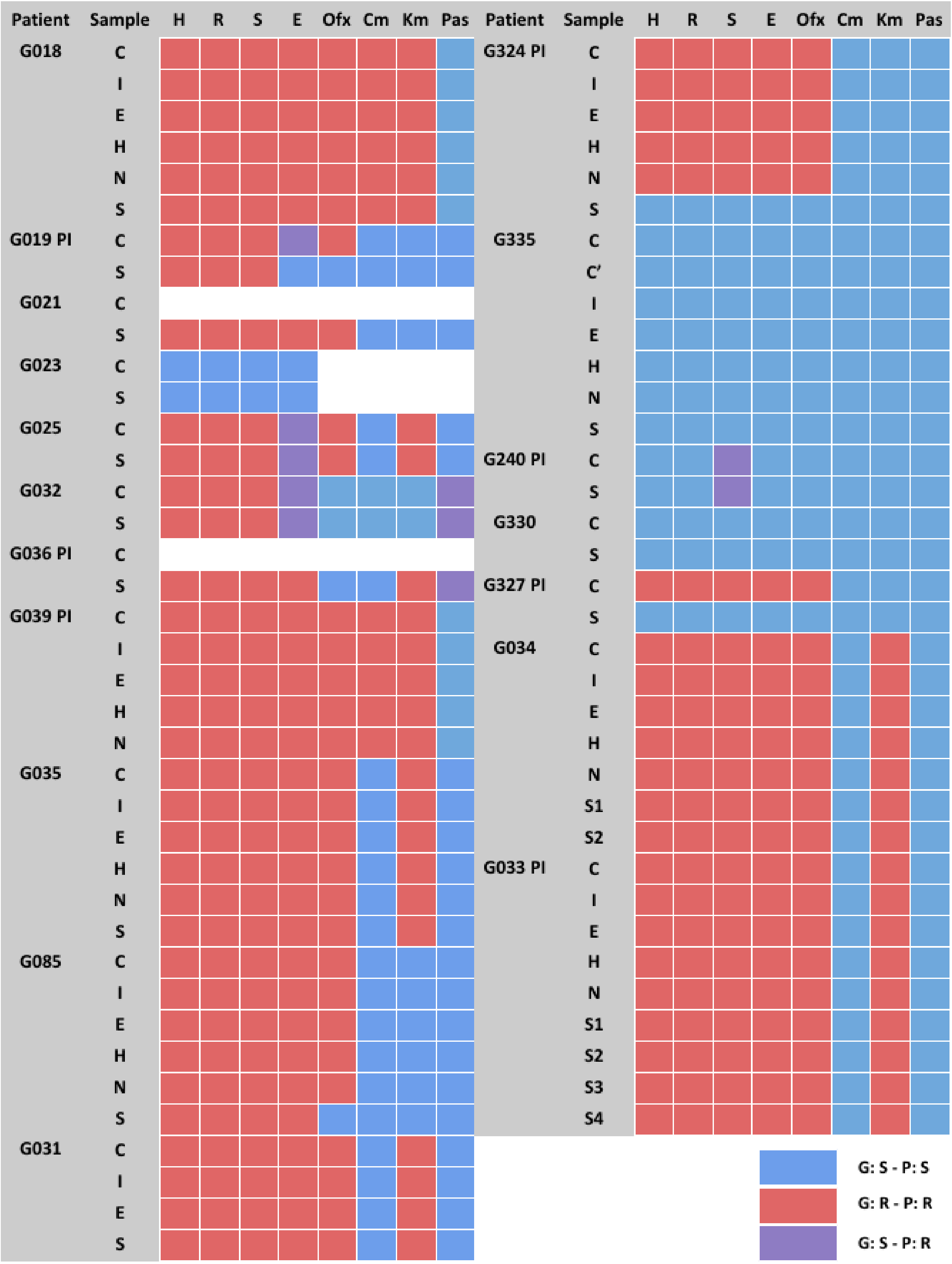

Supplementary Figure 7. Comparison matrix of resistance prediction based on genotype and drug susceptibility testing (DST). Legend shows three different colors to explain matches and mismatches between them. Blue means susceptibility match, red means resistance match and purple means disagreement in which genomic data failed to predict phenotypic resistance.
