# Supplementary material for: Genomic analyses of Mycobacterium tuberculosis from human lung resections reveal a high frequency of polyclonal infections
Source: Nat Commun. 2021 May 11;12:2716. doi: 10.1038/s41467-021-22705-z (PMC8113332; doi:10.1038/s41467-021-22705-z)
Supplement: Supplementary file 3 — Description of Additional Supplementary Files [file 41467_2021_22705_MOESM3_ESM.pdf]

Description of Additional Supplementary Files

File Name: Supplementary Data 1
Description: List of accession numbers and patient related metadata from this study.

File Name: Supplementary Data 2
Description: List of discarded genes for variant calling due to mapping errors.
