# Supplementary material for: Genomic analyses of Mycobacterium tuberculosis from human lung resections reveal a high frequency of polyclonal infections
Source: Nat Commun. 2021 May 11;12:2716. doi: 10.1038/s41467-021-22705-z (PMC8113332; doi:10.1038/s41467-021-22705-z)
Supplement: Supplementary file 6 — Reporting Summary [file 41467_2021_22705_MOESM6_ESM.pdf]

nature research

Corresponding author(s): Iñaki Comas (icomas@ibv.csic.es)

Last updated by author(s): Mar 15, 2021

Reporting Summary

Nature Research wishes to improve the reproducibility of the work that we publish. This form provides structure for consistency and transparency in reporting. For further information on Nature Research policies, see our Editorial Policies and the Editorial Policy Checklist.

Statistics

For all statistical analyses, confirm that the following items are present in the figure legend, table legend, main text, or Methods section.

n/a	Confirmed	
☐	☒	The exact sample size (*n*) for each experimental group/condition, given as a discrete number and unit of measurement
☒	☐	A statement on whether measurements were taken from distinct samples or whether the same sample was measured repeatedly
☐	☒	The statistical test(s) used AND whether they are one- or two-sided<br>*Only common tests should be described solely by name; describe more complex techniques in the Methods section.*
☒	☐	A description of all covariates tested
☒	☐	A description of any assumptions or corrections, such as tests of normality and adjustment for multiple comparisons
☐	☒	A full description of the statistical parameters including central tendency (e.g. means) or other basic estimates (e.g. regression coefficient) AND variation (e.g. standard deviation) or associated estimates of uncertainty (e.g. confidence intervals)
☐	☒	For null hypothesis testing, the test statistic (e.g. *F*, *t*, *r*) with confidence intervals, effect sizes, degrees of freedom and *P* value noted<br>*Give P values as exact values whenever suitable.*
☒	☐	For Bayesian analysis, information on the choice of priors and Markov chain Monte Carlo settings
☒	☐	For hierarchical and complex designs, identification of the appropriate level for tests and full reporting of outcomes
☒	☐	Estimates of effect sizes (e.g. Cohen's *d*, Pearson's *r*), indicating how they were calculated

Our web collection on statistics for biologists contains articles on many of the points above.

Software and code

Policy information about availability of computer code

Data collection	No software was used for data collection.
Data analysis	Reads preprocessing: fastp 0.12.5<br>Taxonomic filtering: Kraken 0.10.5-beta<br>Reads mapping: bwa 0.7.12-r1039<br>Alignment processing: samtools 1.3.1 and Picard 2.18.0-SNAPSHOT<br>Variant calling: varscan2 2.3.7, GATK 3.8-1-0-gf15c1c3ef, lofreq 2.1.3.1<br>Phylogeny construction: MEGAv 10.0.5, IQ-TREE v1.6.11<br>Synthetic read simulation: ART, Q version 2.5.8<br>Plotting: ggplot v3.1.1, affycoretools v1.50.6<br><br>Custom Python scripts were used for data processing and analysis, and are available at https://gitlab.com/tbgenomicsunit/georgia-polyclonal

For manuscripts utilizing custom algorithms or software that are central to the research but not yet described in published literature, software must be made available to editors and reviewers. We strongly encourage code deposition in a community repository (e.g. GitHub). See the Nature Research guidelines for submitting code & software for further information.

Data

Policy information about availability of data

All manuscripts must include a data availability statement. This statement should provide the following information, where applicable:

- Accession codes, unique identifiers, or web links for publicly available datasets
- A list of figures that have associated raw data
- A description of any restrictions on data availability

Sequencing files for all patients in the study are available at Bioprojects PRJNA480888 and PRJNA318002. Additional data on patients is available at TB Portals (https://data.tbportals.niaid.nih.gov). Resistance prediction databases are available at: PhyResSE; https://bioinf.fz-borstel.de/mchips/phyresse and ReSeqTB; https://platform.reseqtb.org.

Field-specific reporting

Please select the one below that is the best fit for your research. If you are not sure, read the appropriate sections before making your selection.

[x] Life sciences   [ ] Behavioural & social sciences   [ ] Ecological, evolutionary & environmental sciences

For a reference copy of the document with all sections, see nature.com/documents/nr-reporting-summary-flat.pdf

Life sciences study design

All studies must disclose on these points even when the disclosure is negative.

Sample size	No sample size calculation was performed. Patients eligible for surgical resection of pulmonary tuberculosis are uncommon, and only 20 patients were recruited during the whole study period.
Data exclusions	No data was excluded from the analysis.
Replication	Due to the origin of the samples - surgical resection - it is difficult to evaluate reproducibility.
Randomization	Allocation was not random but based on the persistence of abnormal lung lesions (predominantly cavities) identified on chest X-ray despite good treatment adherence. This included both susceptible and drug-resistant tuberculosis cases.
Blinding	Blinding was not possible due to the nature of the surgical samples. Patient eligibility was restricted to availability, processing of surgical samples is different from sputa and the goal of the study was to compare between the two types of sampling in different patients.

Reporting for specific materials, systems and methods

We require information from authors about some types of materials, experimental systems and methods used in many studies. Here, indicate whether each material, system or method listed is relevant to your study. If you are not sure if a list item applies to your research, read the appropriate section before selecting a response.

Materials & experimental systems

n/a	Involved in the study	
[x]	[ ]	Antibodies
[x]	[ ]	Eukaryotic cell lines
[x]	[ ]	Palaeontology and archaeology
[x]	[ ]	Animals and other organisms
[ ]	[x]	Human research participants
[ ]	[x]	Clinical data
[x]	[ ]	Dual use research of concern

Methods

n/a	Involved in the study	
[x]	[ ]	ChIP-seq
[x]	[ ]	Flow cytometry
[x]	[ ]	MRI-based neuroimaging

Human research participants

Policy information about studies involving human research participants

Population characteristics	Patients of any age and sex undergoing therapeutic surgery for their Pulmonary Tuberculosis at NCTLD in Tbilisi, Georgia indicated as per clinical routine. Population characteristics are available in Supplementary Data 1.
Recruitment	Recruitment at the hospital via informed consent before therapeutic surgical resection. Most of the patients undergoing surgery had already been diagnosed at least as MDR-TB and did not respond to treatment. This population is characterised by substantial exposure to multiple antibiotics, a higher frequency of prior TB disease, the frequent presence of cavitary disease (associated with poor antibiotic penetration), and sometimes prolonged hospitalisation that can increase the risk of superinfection.
Ethics oversight	Ethical approval for the study (#892/01-17) was obtained from the Institutional Review Board (IRB) of the National Center for Tuberculosis and Lung Diseases (NCTLD), Georgia.

Note that full information on the approval of the study protocol must also be provided in the manuscript.

Clinical data

Policy information about clinical studies

All manuscripts should comply with the ICMJEguidelines for publication of clinical research and a completedCONSORT checklist must be included with all submissions.

Clinical trial registration	Trial number NCT02715271 at ClinicalTrials.gov
Study protocol	https://clinicaltrials.gov/ProvidedDocs/71/NCT02715271/Prot_ICF_000.pdf
Data collection	The approved protocol includes lesions collection during therapeutic surgery, as well as the collection of demographic data, clinical features and analytical data of the patients included in the study, data on their evolution pre and post-surgery, complications and survival. Clinical data were collected at NCTLD (Georgia) between January 2013 and March 2018.
Outcomes	1. Description of Clinical and Epidemiological Data of the TB patients enrolled.<br>2. Description of the Histopathological characteristics of the TB lesions of TB patients enrolled.
